# Distinctive pattern of left–right asymmetry of ovarian benign teratomas in Chinese population: a 12-year-long cross-sectional study

**DOI:** 10.1007/s00404-020-05864-0

**Published:** 2021-01-11

**Authors:** Xiaoqing He, Xiaoya Zhao, Xiaofeng Wang, Guiling Liang, Hang Qi, Chenfeng Zhu, Zhen Huang, Jian Zhang

**Affiliations:** 1 Department of Obstetrics and Gynecology, School of Medicine, International Peace Maternity and Child Health Hospital, Shanghai Jiaotong University, Shanghai, China; 2 Shanghai Key Laboratory Embryo Original Diseases, Shanghai, China; 3 Shanghai Municipal Key Clinical Specialty, Shanghai, China

**Keywords:** Ovarian teratoma, Left–right asymmetry, Ovarian torsion, Recurrence

## Abstract

**Purpose:**

Given the lack of research on the left–right asymmetry of ovarian teratoma among Chinese patients, this study aimed to determine the lateral distribution and related clinical characteristics of Chinese ovarian teratoma patients treated at a single center.

**Methods:**

We conducted a cross-sectional study of surgical patients pathologically diagnosed with ovarian teratomas in the gynecology inpatient department of the International Peace Maternity and Child Health Hospital in Shanghai between July 2006 and July 2018.

**Results:**

Of the 4417 patients with ovarian teratoma, 3835 were finally analyzed. There were 2030 (53.24%) cases of right-sided benign ovarian teratoma versus 1783 (46.76%) cases of left-sided benign teratoma (*P* < 0.001). The recurrence rate of benign ovarian teratoma was 4.2%; recurrence occurred more often on the left side (left vs. right = 55 vs. 45%, *P* = 0.033). Compared with the right-sided ovarian teratoma patients, left-sided ones had significantly high recurrence risk (OR 1.430; 95% CI 1.03–1.99). The rate of ovarian torsion in patients with ovarian mature cystic teratomas (MCTs) during intrauterine pregnancy was 3.17 versus 1.72% in non-pregnant MCT patients (*P* = 0.049). For those MCT patients with intrauterine pregnancy, ovarian torsion occurs more often on the right side (left vs. right = 16.67 vs. 83.33%, *P* = 0.028).

**Conclusion:**

This study confirms a distinctive right-side dominance of benign ovarian teratomas. Compared with the right side, recurrent ovarian teratomas occur more often on the left side, requiring close follow-up. Intrauterine pregnancy may increase the risk of ovarian torsion, particularly on the right side, in MCT patients.

## Background

Ovarian teratomas (OTs) are the most common neoplasms of the ovary, constituting 10–20% of all ovarian tumors in adults and almost half of all ovarian tumors in children, and these tumors originated from three layers, namely ectoderm, mesoderm, and endoderm [1, 2]. Generally, OTs could be classified as mature cystic teratomas (MCTs), monodermal teratomas, ovarian immature teratomas (OITs), etc. [3, 4]. MCTs are the most common subtypes found in the ovary, which account for approximately 95% of all germ cell tumors [5, 6].

Most OTs are asymptomatic and usually discovered during pelvic examination, and complications include ovarian torsion, malignant degeneration, tumor rupture, ovarian vein thrombophlebitis, etc., resulting in sepsis and thrombosis of the inferior vena cava and renal veins [7–9]. Inferior vena cava thrombosis can cause serious complications such as pulmonary embolism that can be life-threatening [9, 10]. Another rare but serious complication of OTs is anti-N-methyl-d-aspartate receptor (NMDAR) encephalitis, which results in a characteristic syndrome presenting with prominent psychiatric symptoms or, less frequently, memory deficits, followed by a rapid decline of the level of consciousness, central hypoventilation, seizures, involuntary movements, and dysautonomia [11].

Ismail et al. indicated that 80–90% of ovarian thrombosis occurs on the right ovary, which accounts for 25% of pulmonary embolism and 5% of deaths in complicated cases, and the right-side dominance of ovarian MCTs may improve such risk [9]. However, the lateral distribution of OTs remains controversial to date. Previous studies suggested that right-side MCTs account for 43.5–72.2% [9,12–16] with a bilateral incidence of 8–15%. Paolo Vercellini found no significant difference in the left–right distribution of MCTs. Mumtaz Khan et al. [13] found the left-side predominance in the MCTs (left vs. right = 64.3 vs. 35.7%). Few studies have focused on the left–right distributions of OITs and monodermal teratomas. Understanding the left–right asymmetry of OTs could help us develop a follow-up plan for patients and prevent OT recurrence and ovarian torsion caused by OTs. However, results of all aforementioned studies were based on a relatively small sample size and different populations, focused only on MCTs, and barely have information of the lateral distribution of OTs in China. Given the lack of research on the right-left asymmetry of OT among Chinese patients, this hospital-based cross-sectional study aimed to determine the lateral distribution and related clinical characteristics of Chinese OT patients in a single center.

## Materials and methods

### Study design and participants

This hospital-based cross-sectional study was conducted in the gynecology inpatient department of the International Peace Maternity and Child Health Hospital in Shanghai, which is a maternity hospital with more than 10,000 gynecological operations per year. Women pathologically diagnosed with teratomas [38] between July 2006 and July 2018 were included, and their previous OTs during this period were counted as gynecological and surgical history. To avoid the effect of racial difference, non-Chinese patients were left out. Patients diagnosed with non-OTs or OT patients with non-ovarian malignant tumors were excluded. Patients with only one ovary or diagnosed with bilateral OTs were ruled out. OT patients with malignant ovarian epithelial tumors or other malignant ovarian germ cell tumors were also excluded.

This study was approved by the institutional review board of the International Peace Maternity and Child Health Hospital in Shanghai, China (GKLW-2018-41), in accordance with the ethical standards of the Declaration of Helsinki. In addition, participant’s information was kept confidential.

### Data collection

We obtained all patients’ data retrospectively from the EMR system of the hospital. Data included demographic characteristics (such as age and nationality), gynecological and surgical history (such as number of previous oophorectomy, side of last ovarian cyst, treatment of last ovarian cyst), clinical features (such as main symptoms and complications), surgery record, and pathological result.

In our study, OTs could be classified as benign OTs and malignant teratomas. Benign OTs are usually categorized by the number of germ layers involved: MCTs (derived from at least two of the three germ cell layers) and monodermal OTs (derived from predominantly or solely of one tissue type, including struma ovarii, carcinoid tumors and neural tumors) [3]. Malignant OTs could be categorized by the same way into OITs (originated from more than one germ layer) and malignant monodermal OTs (originated from one germ layer).

### Statistical analysis

Pearsonʼs χ^2^ test was used to explore the association between clinical characteristics and lateralities of OTs. One-way analysis of variance was performed to compare the average age and tumor diameter among different categories, age groups, and lateralities of OTs. Linear regression calculated the *P *value for trend. The binominal test was used to investigate the left–right asymmetry of OTs in different categories and age groups, proposing the null hypothesis that the probability of OTs in left/right ovary is 50%, against the alternative hypothesis that the probability of OTs in left/right ovary is not 50%. OR and 95% CI were obtained for variables in the multivariate logistic regression model. All statistical analyses were performed using R 3.6.1 and IBM SPSS statistics 25. All *P* values less than 0.05 were considered statistically significant.

## Results

### Overall lateral distribution pattern of OT

**Fig. 1:**
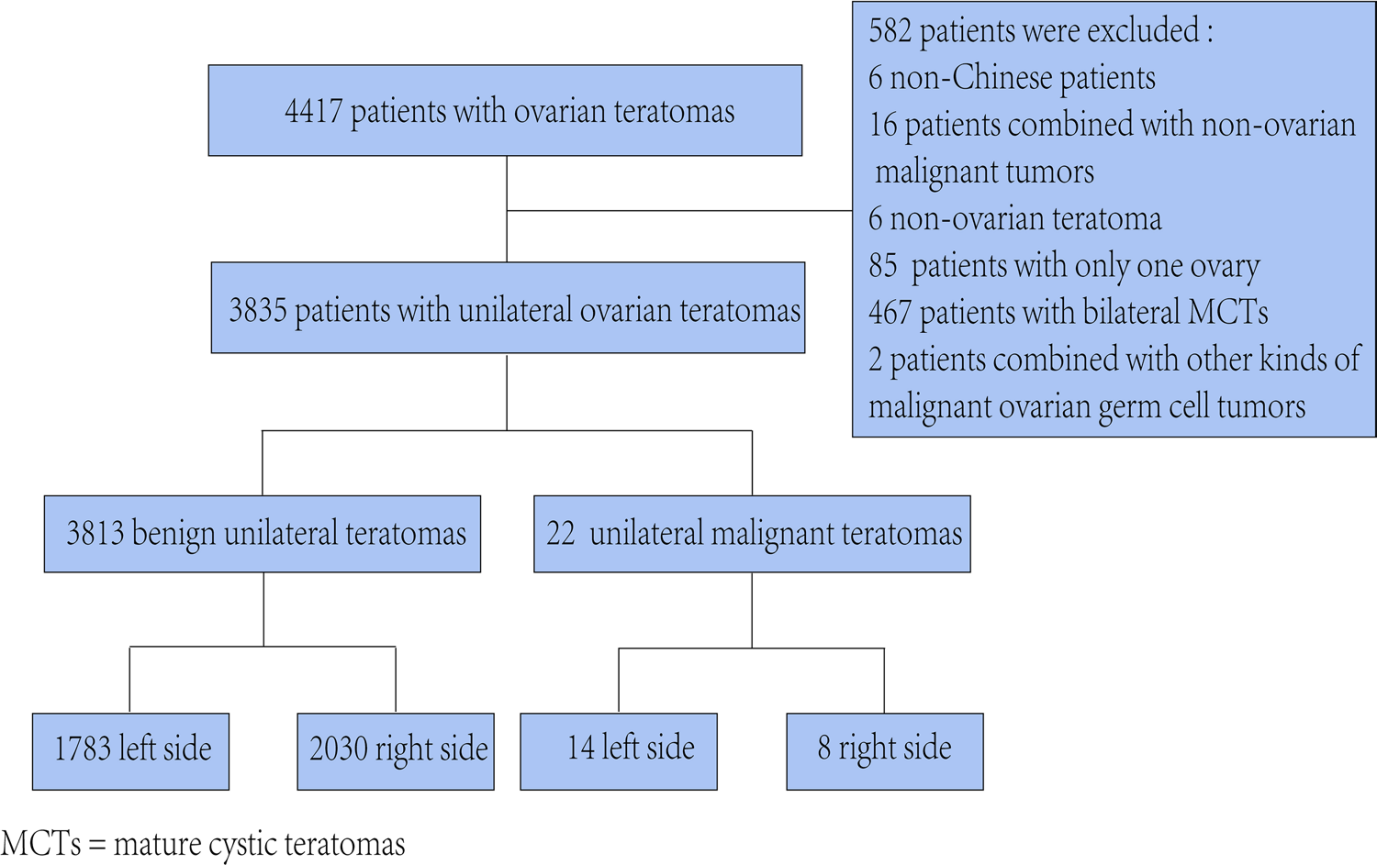
Flowchart of the cross-sectional study

As shown in the flowchart (Fig. 1), we identified 4417 hospitalized surgical patients diagnosed with OTs between July 2006 and July 2018, of which 582 patients were excluded; finally, a total of 3835 patients with unilateral OTs were analyzed. There was a right-side dominance of benign ovarian OT distribution (right vs. left = 2030 (53.24%) vs. 1783 (46.76%), *P* < 0.001). Among benign OTs, MCTs showed distribution asymmetries on both sides (*P* < 0.001); meanwhile, left–right distribution of monodermal teratomas was comparable. There was more malignant OTs on the left side than on the right side, but no statistically significant difference was found (left vs. right = 14 (63.64%) vs. 8 (36.36%), *P* = 0.286), neither in OITs nor in malignant monodermal teratomas (Table 1).

**Table 1:** Classification of unilateral ovarian teratoma

	Total	Left		Right		*P* value^a^
		*n*	(%)	*n*	(%)	
Benign ovarian teratoma	3813	1783	(46.76)	2030	(53.24)	< 0.001
MCT	3745	1752	(46.78)	1993	(53.22)	< 0.001
Monodermal teratoma^b^	68	31	(45.59)	37	(54.41)	0.545
Malignant ovarian teratoma	22	14	(63.64)	8	(36.36)	0.286
OIT	19	11	(57.89)	8	(42.11)	0.648
Malignant monodermal teratoma^c^	3	3	(100.00)	0	(0.00)	0.250
Total	3835	1797	(46.86)	2038	(53.14)	< 0.001

*MCT* ovarian mature teratoma, *OIT *ovarian immature teratoma

^a^Binomial test

^b^Of the 68 cases of benign monodermal teratoma, 66 cases had struma ovarii and 2 cases had epidermoid cysts

^c^Three cases of malignant monodermal teratomas were malignant struma ovarii

### Lateral distribution pattern by clinical characteristics

#### Main symptoms

As shown in Table 2, approximately 84% of benign OTs are asymptomatic, of which MCTs occur in 84.11% and monodermal teratomas in 77.94% of the patients. The main symptoms (abdominal mass, abdominal discomfort, acute abdominal pain, and compression symptom) in MCTs and monodermal teratomas were similar on both sides (MCTs, *P* = 0.289; monodermal teratomas, *P* = 0.823), and three patients with left-sided MCTs developed neurological symptoms. The main symptoms of malignant OTs were abdominal discomfort and abdominal mass, without significant difference on the two sides (*P* = 0.673).

**Table 2:** Clinical characteristics in unilateral ovarian teratoma

	Total	Left side		Right side		*P* value
	*n*	*n*	(%)	*n*	(%)	
Main symptom of unilateral ovarian teratomas						
Benign ovarian teratomas						
MCT						
Asymptomatic	3150	1485	(47.14)	1665	(52.86)	0.289^a^
Abdominal mass	56	23	(41.07)	33	(58.93)	
Abdominal distension/chronic abdominal pain	390	171	(43.85)	219	(56.15)	
Acute abdominal pain	73	33	(45.21)	40	(54.79)	
Constipation/frequent micturition	73	37	(50.68)	36	(49.32)	
Mental and nervous symptoms	3	3	(100.00)	0	(0.00)	
Monodermal teratomas						0.823^a^
Asymptomatic	53	25	(47.17)	28	(52.83)	
Abdominal mass	3	2	(66.67)	1	(33.33)	
Abdominal distension/chronic abdominal pain	5	2	(40.00)	3	(60.00)	
Acute abdominal pain	3	1	(33.33)	2	(66.67)	
Constipation / frequent micturition	4	1	(25.00)	3	(75.00)	
Malignant ovarian teratomas						
OIT						
Asymptomatic	4	3	(75.00)	1	(25.00)	0.673^a^
Abdominal mass	5	3	(60.00)	2	(40.00)	
Abdominal distension/chronic abdominal pain	2	1	(50.00)	1	(50.00)	
Acute abdominal pain	1	1	(100.00)	0	(0.00)	
Constipation/frequent micturition	5	2	(40.00)	3	(60.00)	
Repeated fever of unknown origin	1	0	(0.00)	1	(100.00)	
Abdominal uterine bleeding	1	1	(100.00)	0	(0.00)	
Malignant monodermal teratomas						
Asymptomatic	3	3	(100.00)	0	(0.00)	NA
Age of patients with unilateral ovarian teratomas (mean ± SD, year)						
Benign ovarian teratomas	34.05 ± 10.49	34.17 ± 10.50		33.94 ± 10.49		0.495^b^
MCT	33.90 ± 10.40	34.06 ± 10.46		33.77 ± 10.36		0.395^b^
Monodermal teratomas	41.97 ± 12.31	40.58 ± 11.22		43.14 ± 13.20		0.398^b^
Malignant ovarian teratomas	30.32 ± 13.41	32.36 ± 15.52		26.75 ± 8.33		0.358^b^
OIT	26.26 ± 8.85	25.91 ± 9.60		26.75 ± 8.33		0.845^b^
Malignant monodermal teratomas	56.00 ± 6.25	56.00 ± 6.25		0		NA
Diameter of unilateral teratomas (mean ± SD, cm)						
Benign ovarian teratomas	5.88 ± 2.62	5.91 ± 2.55		5.85 ± 2.68		0.499^b^
MCT	5.86 ± 2.60	5.89 ± 2.53		5.82 ± 2.65		0.447^b^
Monodermal teratomas	7.03 ± 3.40	6.84 ± 3.26		7.20 ± 3.56		0.670^b^
Malignant ovarian teratomas	15.82 ± 8.44	15.64 ± 9.30		16.17 ± 7.39		0.906^b^
OIT	17.93 ± 7.71	19.35 ± 8.17		16.17 ± 7.39		0.481^b^
Malignant monodermal teratomas	6.00 ± 2.65	6.00 ± 2.65		0		NA
Recurrence of benign teratomas, *n*/*N *(%) ^c^	160/3813 (4.20)	88/1783 (4.94)	(55.00)	72/2030 (3.55)	(45.00)	**0.033**^**a**^
MCT	150/3745 (4.01)	83/1752 (4.74)	(55.33)	67/1993 (3.36)	(44.67)	**0.037**^**a**^
Monodermal teratomas	10/68 (14.71)	5/31 (16.13)	(50.00)	5/37 (13.51)	(50.00)	0.762^a^

*MCT *mature cystic teratomas, *OIT* ovarian immature teratomas, *malignant teratoma *19 cases OIT + 3 cases malignant struma ovarii

^a^Pearsonʼs χ^2^ test

^b^One-way analysis of variance (ANOVA)

^c^Recurrence is defined as one or more occurrence of previous ovarian teratomas

#### Age at diagnosis

**Fig. 2:**
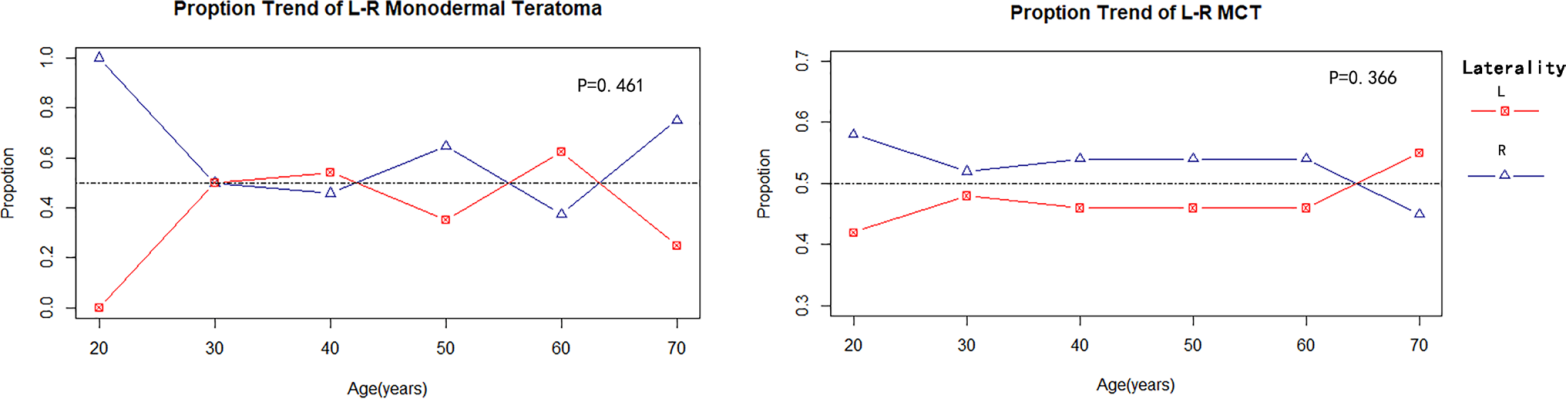
Line chart displaying the left–right (L–R) distribution of unilateral mature cystic teratomas (MCT) and monodermal teratomas among different age groups

Patients’ age at diagnosis ranged from 8 to 82 years. In Table 2, the average age (mean ± standard deviation (SD), years) of patients with benign OTs (34.05 ± 10.49 years) was similar to that of patients with malignant OTs (30.32 ± 13.41 years) (*P* = 0.097). Among benign OT patients, on average, MCT patients (33.90 ± 10.40 years) were younger than those of monodermal teratoma patients (41.97 ± 12.31 years) (*P* < 0.001). Among malignant OT patients, OIT patients (26.26 ± 8.85 years) were apparently younger than malignant monodermal OT patients (56.00 ± 6.25 years) (*P* < 0.001). However, the average patient age on diagnosis of both benign and malignant OTs, as well as MCTs, monodermal OTs, OITs, and malignant monodermal OTs, on the two sides were similar (Table 2). Figure 2 indicates no remarkable difference in the left–right distribution of age in neither MCT nor monodermal teratoma patients. Given the small sample size of malignant OT patients, we failed to further explore the lateral distribution pattern by subgroups of age.

#### Maximum diameter of OTs

**Fig. 3:**
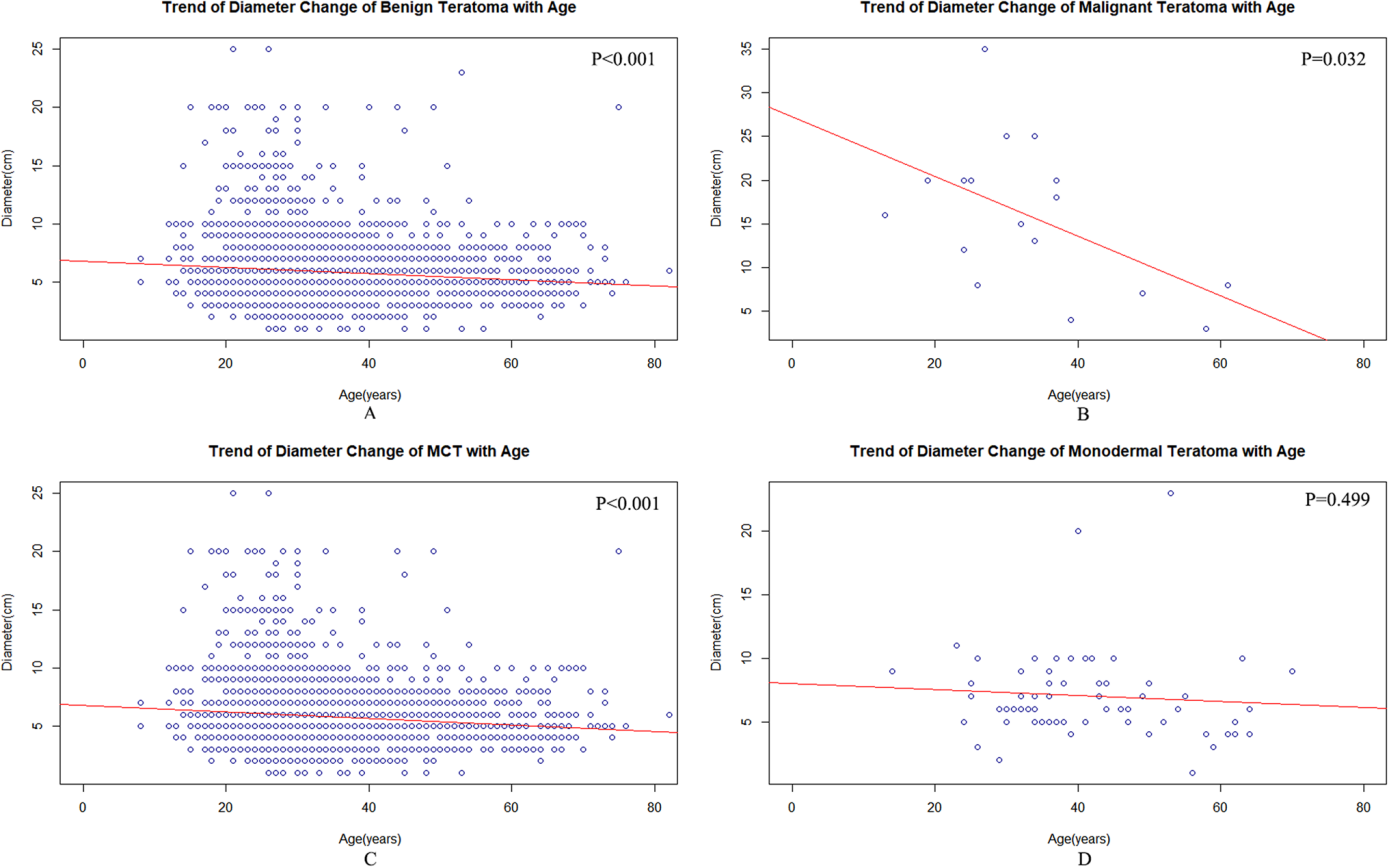
Scatter plots indicate the relation between age and diameter of ovarian mass among benign teratomas (**a**), malignant teratomas (**b**), mature cystic teratomas (**c**), monodermal teratomas (**d**)

The average diameter (mean ± SD, cm) of benign OTs (5.88 ± 2.62 cm) was apparently smaller than that of malignant OTs (15.82 ± 8.44 cm) (*P* < 0.001). Among benign OTs, the average diameter of MCTs (5.86 ± 2.60 cm) was smaller than that of monodermal teratomas (7.03 ± 3.40 cm) (*P* < 0.001). Among malignant OTs, the average diameter of OITs (17.93 ± 7.71 cm) was more significant than that of malignant monodermal teratomas (6.00 ± 2.65) (*P* = 0.021). Furthermore, the diameter showed a significant downtrend along with age in both benign and malignant teratomas (Fig. 3a, b). Among benign teratomas, compared with MCTs, monodermal teratomas did not show a significant downtrend in diameter (Fig. 3c, d). Nonetheless, the average diameter of teratomas was similar in the two sides of the aforementioned categories (Table 2).

### Association of lateral distribution pattern and clinical outcomes

#### Recurrence of benign OTs

Table 2 shows that the recurrence rate of benign OTs was 4.2%, recurrence occurs more often on the left side (left vs. right = 55 vs. 45%, *P* = 0.033). Among benign OTs, monodermal teratomas recur more often than MCTs (14.71 vs. 4.01%, *P* < 0.001, Pearsonʼs χ^2^ test). For MCTs, recurrence occurs more often on the left side (left vs. right = 55.33 vs. 44.67%, *P* = 0.037), and the multivariate logistic regression analysis also indicated that the left side was significantly related to recurrence (odds ratio (OR) 1.430; 95% confidence interval (CI) 1.029–1.986).

### Complications

#### Anti-NMDAR encephalitis

Three MCT patients developed neurological symptoms and were finally diagnosed with anti-NMDAR encephalitis, which all developed on the left side. They were treated with ventilatory and immunotherapy, and oophorocystectomies were performed as soon as their vital signs were stable.

#### Ovarian torsion

The total ovarian torsion rate was 1.93% (benign vs. malignant = 1.89 vs. 9.09%, *P* = 0.066, Fisher’s exact test), without left–right asymmetry (Table 3). The rate of ovarian torsion in MCT patients with intrauterine pregnancy (none of the monodermal or malignant teratoma) was 3.17%, which was higher than the 1.72% of non-pregnant MCT patients (*P* = 0.049). Moreover, compared with the absence of left–right asymmetry of ovarian torsion among non-pregnant MCT patients, among the 379 MCT patients with intrauterine pregnancy, ovarian torsion was more likely to occur on the right side (left vs. right = 16.67 vs. 83.33%, *P* = 0.028, Table 3).

**Table 3:** Ovarian torsion of unilateral teratoma

	Left side		Right side		*P* value
	*n*/*N* (%)	(%)	*n/N* (%)	(%)	
Ovarian torsion^a^	31/1797 (1.73)	(41.89)	43/2038 (2.11)	(58.11)	0.387^b^
Benign ovarian teratoma	29/1783 (1.63)	(40.28)	43/2030 (2.12)	(59.72)	0.266^b^
MCT	27/1752 (1.54)	(38.57)	43/1993 (2.16)	(61.43)	0.165^b^
Monodermal teratoma	2/31 (6.45)	(100.00)	0	(0.00)	NA
Malignant ovarian teratoma	2/14 (4.29)	(100.00)	0	(0.00)	NA
Ovarian torsion of teratomas combined with intrauterine pregnancy^c^					
MCT	2/181 (1.11)	(16.67)	10/198(5.05)	(83.33)	**0.028**^**b**^

*MCT *mature cystic teratoma, *SD *standard deviation, *malignant teratoma *ovarian immature teratoma + malignant monodermal teratoma

^a^Two cases of ovarian torsion in malignant teratomas were both ovarian immature teratomas

^b^Pearsonʼs χ^2^ test

^c^Zero case of ovarian torsion combined with intrauterine pregnancy in malignant teratoma or monodermal teratoma

## Discussion

In this study, we found a distinctive pattern of right-side dominance of benign OTs. Moreover, malignant OTs showed no significant difference in the distribution between the two sides. Compared with right-sided benign OT patients, the left-sided ones had a significantly increased risk of recurrence. MCT patients with intrauterine pregnancy more often experienced ovarian torsion than non-pregnant MCT patients (3.17 vs. 1.72%, *P* = 0.049). Among MCT patients with intrauterine pregnancy, ovarian torsion occurs more often on the right side (left vs. right = 16.67 vs. 83.33%, *P* = 0.028).

In this study, there was a right-side dominance of benign OTs among Chinese women, as was previously reported by Ismail et al. and Khan et al. [9, 13]. The left–right asymmetry of organ positioning and morphologies are common in humans, which are established during embryonic development and under genetic control [17]. Roychoudhuri et al.’s [18] study of 306,214 patients has shown that the left–right asymmetry of many cancers may be explained by the larger organ size on that side. Moreover, Roychoudhuri et al. [19] reflected a right-side predominance in 288 cases of ovarian germ cell cancers; however, they did not report ovarian tumor size and category information. Another study has also shown a right-side dominance (right at 50.1% vs. left at 35.1%) among 427 cases of ovarian dysgerminoma. Compared with higher occurrence of left-sided malignant OTs in our study, it was reasonable to assume that difference may exist in the left–right distribution among different categories of ovarian germ cell cancers. This discrepancy in left–right distribution of ovarian germ cell cancers between our study and previous studies might be due to the small sample size or use of different categories of ovarian germ cell cancers. This is an interesting topic worth exploring in the future.

Furthermore, Møller et al. [20] indicated that the incidence of germ cell cancer increased sharply around the age of onset of puberty and decreased with increase in age, which is in line with our result. Thus, it is reasonable to speculate that the right-side dominance of OT may be associated with the left–right asymmetry of follicles and ovulation. Kaku et al. [1]. suggested that OTs originate from post-meiotic oocytes or ova; that is, the higher the number post-meiotic oocytes or ova, the higher the probability of teratomas. Previous studies indicated that the right ovary contains a large number of antral follicles and tended to ovulate more often than the left ovary (54–64%) [21–24]. Therefore, it is plausible to consider that the right-sided predominant follicle population is attributable to the right-side dominance of benign OTs.

However, at present, the genetic, molecular, and other mechanisms of the right predominance of the follicle population are unclear. A possible explanation of the distinctive pattern of left–right asymmetry is the difference in the circulation within the right and left ovaries; as regards venous drainage, right-side veins drain directly into the inferior vena cava; nevertheless, left-sided veins drains via the renal vein into the inferior vena cava.

In our study, there was no left–right asymmetry when considering the average age, neither in benign nor in malignant OTs. The average patient age in the present study is similar to that in a previous study [16]. However, for benign OTs, MCT patients were younger than monodermal teratoma patients on average; for malignant OTs, OIT patients were younger than malignant monodermal teratoma patients on average. It appears that patients with OIT that originated solely from one germ layer were older than those with OIT that originated from at least two germ layers. As no study has compared the age of diagnosis of different categories of OTs, whether the composition of OTs affects average age or not is still unclear.

Contrary to the result of the present study, Chun et al. [12] also suggested that the size of right-side MCTs is more significant than left-sided MCTs because of the small sample size (*n* = 56). Furthermore, it is more plausible to believe that different kinds of OTs may have similar tumor size on two sides. The tumor diameter also showed a significant downtrend along with age, both in benign and malignant OTs, which agreed with the finding of Kim et al. [25] that younger patients have larger OT than older patients.

A previous study suggested that gravidity, parity, cyst size, surgical approach, and rupture during operation did not affect the recurrence of OTs; bilateral teratomas have higher recurrence rate than unilateral teratomas, without comparing left–right side distribution among unilateral teratomas [26]. In the present study, recurrent benign teratomas were more likely to occur on the left side, especially for MCTs, but without some related clinical details. Teratomas with higher recurrence rate should be followed closely.

A systematic review of 174 cases of OT-associated encephalitis showed no significant left–right distribution difference in neither MCTs nor OITs [27]. In our study, the anti-NMDAR encephalitis in three MCT patients all developed on the left side, and it may be occasionally affected by the low incidence of anti-NMDAR encephalitis.

The total torsion rate of MCTs in our study (1.87%) is lower than that in previous studies (3.25–16%) [28], but all these studies were conducted before 1994. Owing to the improvement of medical treatment, majority of OTs may be detected and treated before complications developed.

Previous studies have noted the right-sided predominance of adnexal torsion [29, 30]. In our study, the ovarian torsion rate in the two sides was comparable among different categories of OTs, while MCT patients with intrauterine pregnancy had higher risk for torsional damage, especially on the right side. Yaakov Melcer et al. [31] Indicated that ultrasound findings suggestive of benign cystic teratoma (OR 7.8. 95% CI 1.2–49.4) and location of the ultrasound pathology on the right side (OR 4.7. 95% CI 1.9–11.9) were positively associated with adnexal torsion. Ovarian torsion in pregnant women is a concern; several small case series studies compared the adnexal torsion between pregnant and non-pregnant women [32–35]. A previous study suggested that in vitro fertilization is a risk factor of ovarian torsion; especially in women with induced ovulation, this phenomenon may be persistent in functional ovarian cyst [36]. All these studies did not focus on the lateral distribution of ovarian torsion in pregnant women.

Adnexal torsion results from the increased weight of ovarian cyst, longer length of ovarian and suspensory ligaments, or OT. It is the most common complication of MCTs [28]. The right-sided predominance could be considered a result of the right-sided dominance of OT, longer length of right inherent ligament, and active bowel movements of the right terminal ileum and appendix. Moreover, the colon occupying the left side of the pelvic cavity may prevent left adnexal from twisting [37]. During pregnancy, the rotation to the right was found in two-thirds of the gravid uterus, while the rotation to the left was found in one-third of cases. This may explain the left–right asymmetry of ovarian torsion in pregnant MCT patients with intrauterine pregnancy.

## Limitations

This study has several limitations, as it our analysis primarily relied on retrospective data. We retrieved patients’ clinical information directly from the electronic medical records (EMR) to ensure accuracy and reliability of data, such as age, surgical diagnosis, and surgical history. Given the limitation of the EMR system, we failed to retrieve some potentially important factors, such as hormone concentration and conception method. Moreover, this was a single-center study; thus, it would be challenging to address the detection rate of adnexal torsion. Furthermore, asymptomatic patients may be missed. A multicenter study may reduce these limitations.

## Conclusion

In this study, we found a right-side lateral dominance of benign OT, and malignant OT showed no significant difference between the two sides. Recurrent OT occurs more often on the left side than on the right side; thus, cases should be followed closely. MCT patients with intrauterine pregnancy may have increased risk of ovarian torsion; moreover, among these MCT patients with intrauterine pregnancy, ovarian torsion is more likely to occur on the right side. We should be more cautious about treating abdominal pain in MCT patients with intrauterine pregnancy and MCT patients trying to conceive.

## Data Availability

The datasets used and analyzed during the current study are available from the corresponding author on reasonable request.
